# On‐demand mobile health infrastructures to allow comprehensive remote atrial fibrillation and risk factor management through teleconsultation

**DOI:** 10.1002/clc.23469

**Published:** 2020-10-08

**Authors:** Astrid N. L. Hermans, Rachel M. J. van der Velden, Monika Gawalko, Dominique V. M. Verhaert, Lien Desteghe, David Duncker, Martin Manninger, Hein Heidbuchel, Ron Pisters, Martin Hemels, Laurent Pison, Afzal Sohaib, Arian Sultan, Daniel Steven, Petra Wijtvliet, Robert Tieleman, Dhiraj Gupta, Dobromir Dobrev, Emma Svennberg, Harry J. G. M. Crijns, Nikki A. H. A. Pluymaekers, Jeroen M. Hendriks, Dominik Linz, TeleCheck‐AF investigators

**Affiliations:** ^1^ Department of Cardiology Maastricht University Medical Centre and Cardiovascular Research Institute Maastricht Maastricht The Netherlands; ^2^ Department of Cardiology Radboud University Medical Centre Nijmegen The Netherlands; ^3^ Faculty of Medicine and Life Sciences Hasselt University Hasselt Belgium; ^4^ Heart Center Hasselt Jessa Hospital Hasselt Belgium; ^5^ Hannover Heart Rhythm Center, Department of Cardiology and Angiology Hannover Medical School Hannover Germany; ^6^ Department of Cardiology Medizinische Universität Graz Graz Austria; ^7^ Department of Cardiology Antwerp University Hospital and Antwerp University Antwerp Belgium; ^8^ Department of Cardiology Rijnstate Hospital Arnhem The Netherlands; ^9^ Department of Cardiology Hospital East Limburg Genk Belgium; ^10^ Department of Cardiology St Bartholomew's Hospital, Bart's Health NHS Trust London UK; ^11^ Department of Cardiology King George Hospital London UK; ^12^ Department of Electrophysiology, Heart Center University Hospital Cologne Cologne Germany; ^13^ Department of Cardiology Martini Hospital Groningen The Netherlands; ^14^ Department of Cardiology Liverpool Heart and Chest Hospital Liverpool UK; ^15^ Institute of Pharmacology, West German Heart and Vascular Centre University Duisburg‐Essen Essen Germany; ^16^ Department of Clinical Sciences, Division of Cardiovascular Medicine Karolinska Institutet and Karolinska University Hospital Stockholm Sweden; ^17^ Centre for Heart Rhythm Disorders University of Adelaide and Royal Adelaide Hospital Adelaide Australia; ^18^ College of Nursing and Health Sciences Flinders University Adelaide Australia; ^19^ Department of Biomedical Sciences, Faculty of Health and Medical Sciences University of Copenhagen Copenhagen Denmark

## Abstract

**Background:**

Although novel teleconsultation solutions can deliver remote situations that are relatively similar to face‐to‐face interaction, remote assessment of heart rate and rhythm as well as risk factors remains challenging in patients with atrial fibrillation (AF).

Hypothesis.

Mobile health (mHealth) solutions can support remote AF management.

**Methods:**

Herein, we discuss available mHealth tools and strategies on how to incorporate the remote assessment of heart rate, rhythm and risk factors to allow comprehensive AF management through teleconsultation.

**Results:**

Particularly, in the light of the coronavirus disease 2019 (COVID‐19) pandemic, there is decreased capacity to see patients in the outpatient clinic and mHealth has become an important component of many AF outpatient clinics. Several validated mHealth solutions are available for remote heart rate and rhythm monitoring as well as for risk factor assessment. mHealth technologies can be used for (semi‐)continuous longitudinal monitoring or for short‐term on‐demand monitoring, dependent on the respective requirements and clinical scenarios. As a possible solution to improve remote AF care through teleconsultation, we introduce the on‐demand TeleCheck‐AF mHealth approach that allows remote app‐based assessment of heart rate and rhythm around teleconsultations, which has been developed and implemented during the COVID‐19 pandemic in Europe.

**Conclusion:**

Large scale international mHealth projects, such as TeleCheck‐AF, will provide insight into the additional value and potential limitations of mHealth strategies to remotely manage AF patients. Such mHealth infrastructures may be well suited within an integrated AF‐clinic, which may require redesign of practice and reform of health care systems.

## 1 BACKGROUND

Atrial fibrillation (AF) is the most prevalent cardiac arrhythmia and is associated with increased risk of heart failure, stroke, bleeding, acute coronary syndrome and severe adverse effects of antiarrhythmic drugs, all of which may lead to unplanned cardiovascular hospitalization, morbidity and mortality.^[^ ^1^ ^]^ Management of AF risk factors and monitoring of vital parameters, particularly heart rate and rhythm, are important for the management of AF patients and prevention of AF‐related morbidity.^[^ ^2^ ^]^

Remote monitoring by means of novel technologies provides an opportunity to bring the best standard of care and expertise to the patient rather than the patient having to visit an outpatient clinic. This may be particularly important for the management of patients who live remotely and far away from coordinating medical centers or during catastrophes, including coronavirus disease 2019 (COVID‐19), when attendance of outpatient clinics or traveling to the hospital is not possible or undesirable.^[^ ^3^ ^]^ Although new teleconsultation solutions can produce remote situations that are relatively similar to face‐to‐face interaction, the remote assessment of heart rate and rhythm as well as risk factors remains challenging.

In this review article, we discuss mobile health (mHealth) tools and strategies to remotely monitor heart rate and rhythm and incorporate AF risk factors assessment to allow comprehensive AF management through teleconsultation. Additionally, as a possible solution to improve remote AF care during the COVID‐19 pandemic, we introduce the on‐demand TeleCheck‐AF mHealth approach that allows remote app‐based assessment of heart rate and rhythm around teleconsultations.

## 2 REMOTE HEART RATE AND RHYTHM MONITORING

**FIGURE 1:**
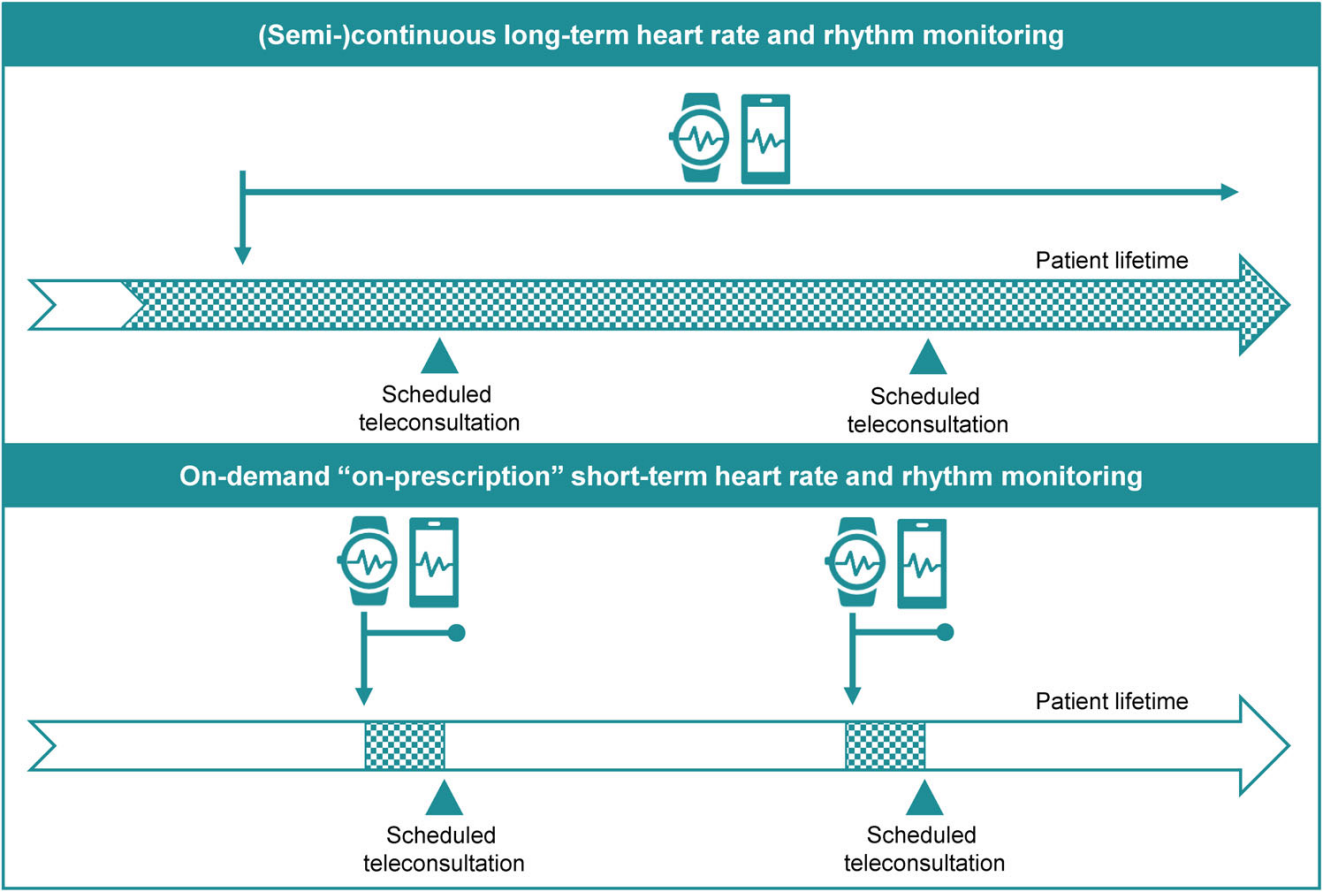
Remote heart rate and rhythm monitoring: (semi‐)continuous longitudinal vs on‐demand

Different mHealth tools are available for remote heart rate and rhythm assessment. Until now, most tools are available within a patient‐initiated paying‐model which, together with the absence of reimbursement, complicates the clinical implementation and guidance of mHealth use by the treating physician. Additionally, the requirements on the way when and how to perform measurements (semi‐continuous vs on‐demand (Figure 1) and electrocardiography (ECG) vs photoplethysmography (PPG), respectively) are critically dependent on the clinical scenario and setting. As this is difficult for patients to judge, a physician‐initiated or at least ‐guided approach appears to be necessary to allow personalized mHealth use and the selection of the right tool for each patient.

**FIGURE 2:**
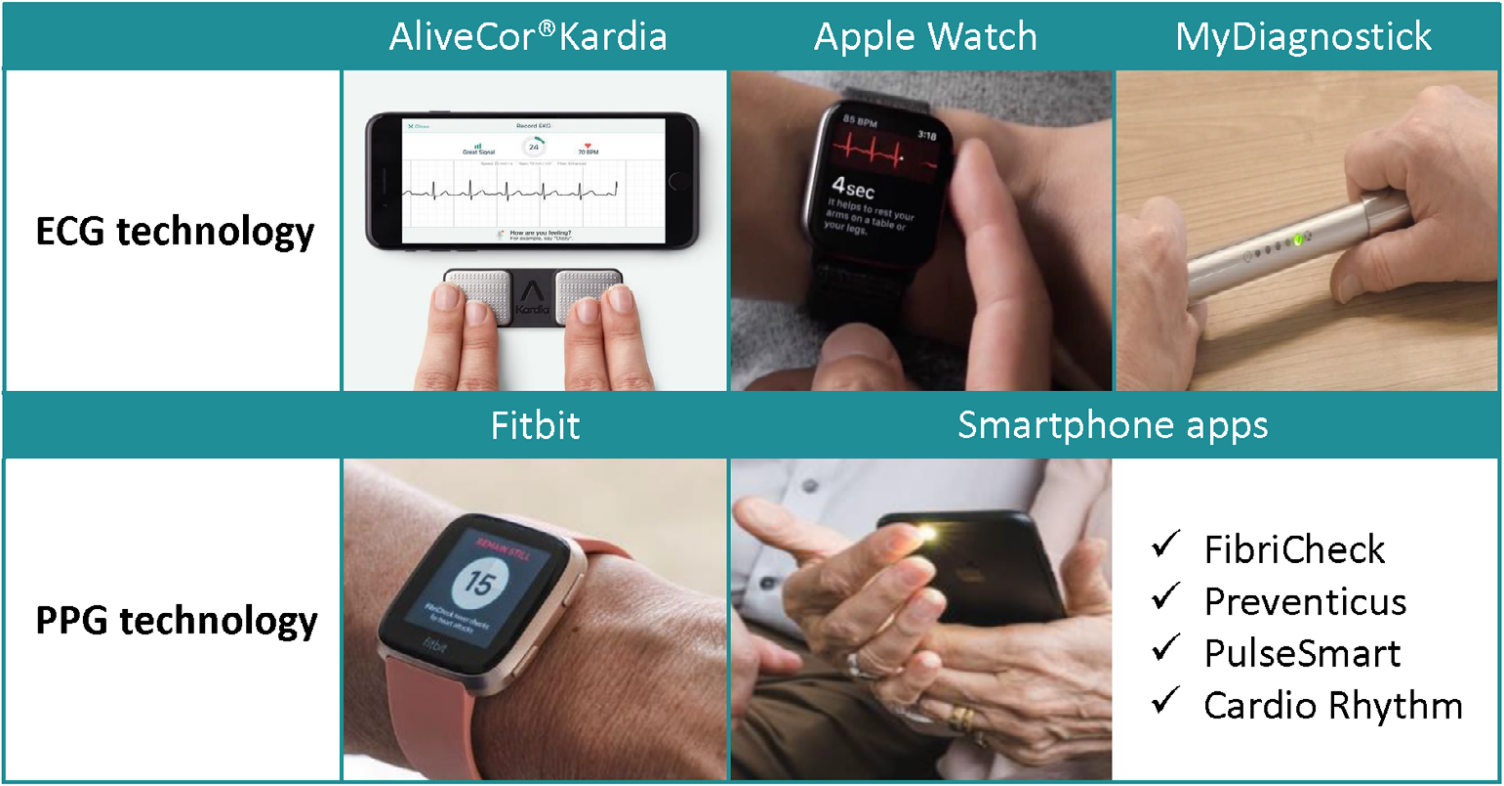
Different mobile health devices

To screen for AF, (semi‐)continuous remote monitoring by cardiac implantable electronic devices (CIEDs), wearables (eg, smart watches), handheld devices (eg, AliveCor, MyDiagnostick, etc.) or app‐based mHealth solutions using PPG technology through the smartphone's built‐in camera (eg, FibriCheck) have been developed and validated (Figure 2). Smartwatches such as Fitbit and Apple Watch, equipped with PPG technology, are commonly used for semi‐continuous heart rate and rhythm monitoring during day and night and have a valuable clinical effect by enabling AF detection in both symptomatic and asymptomatic patients.^[^ ^4^, ^5^, ^6^ ^]^ Most of the devices are CE marked and some of them are connected to secured and certified clouds, allowing remote access of the data by treating physicians or allied healthcare professionals. PPG technology is not sufficient to diagnose AF. Based on the current international AF Guidelines of the European Society of Cardiology (ESC),^[^ ^2^ ^]^ ECG‐documentation of an AF episode is required for diagnosis. As most PPG algorithms are developed for AF screening scenarios, they operate in a high sensitivity mode, resulting in a higher number of false positive recordings.^[^ ^7^ ^]^ Therefore, in the setting of AF screening, PPG‐detected episodes suggestive of AF need to be confirmed by an ECG. Despite crucial differences between PPG and ECG technologies for heart rhythm monitoring, most of the PPG‐based algorithms are validated to detect AF with a high sensitivity and specificity.^[^ ^7^ ^]^ Additionally, there is already some data that PPG technology is nearly as accurate as ECG to detect AF.^[^ ^8^, ^9^ ^]^ In the validation studies for these devices, atrial high rate episodes (AHRE) from CIEDs have not been taken into account. Given the wide availability and low cost, PPG technology may represent an optimal screening tool to detect AF, which can then be confirmed by ECG technology afterwards in a second step.

While PPG technology has limitations to diagnose AF, the wide accessibility and low cost of this technology via smartphone apps makes it an interesting tool for remote heart rate and rhythm monitoring of patients who have already been diagnosed with AF. A temporary pre‐determined on‐demand approach, where heart rate and rhythm information are provided to the health care provider just before a scheduled appointment appears to fulfill most requirements to manage AF patients remotely through teleconsultation. This on‐demand, rather than unfocused long‐term monitoring enables an ideal remote consultation with helpful and crucial well‐timed heart rate and rhythm information available for the treating physician, nurse or allied healthcare professional to steer management of patients with ECG‐documented AF. For an on‐demand mHealth approach, apps can be activated “on prescription” and linked to a secured cloud, which is accessible by the treating physician or nurse. Additionally, simultaneous monitoring of heart rhythm and symptoms provides information about symptom‐rhythm correlation. On‐prescription monitoring increases awareness with the patients who are better prepared for the remote consultation: patients know that symptom and rhythm evaluation is on the agenda during the consultation, together with discussions on necessary treatments. An on‐demand monitoring approach also avoids unnecessary data load, which would require work‐intensive and expensive data management infrastructures.

## 3 REMOTE ASSESSMENT AND MANAGEMENT OF AF RISK FACTORS

**FIGURE 3:**
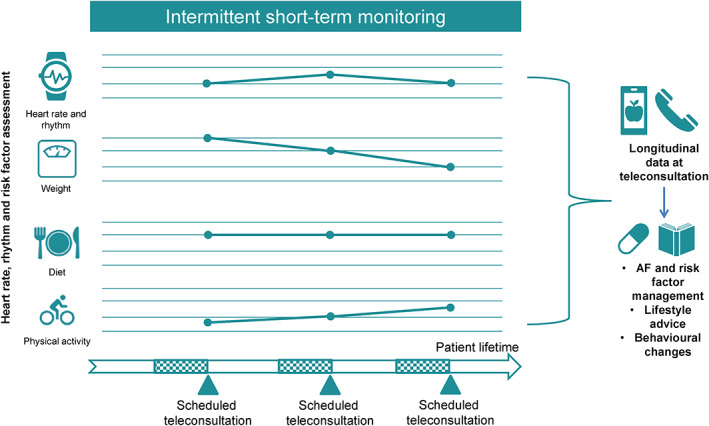
Intermittent short‐term monitoring for longitudinal risk factor assessment. The blue boxes indicate periods of intermittent short‐term monitoring before scheduled teleconsultations

Management of risk factors is an important component of AF treatment. Despite convincing evidence for the need of risk factor management in AF patients,^[^ ^10^ ^]^ it remains unclear, how best to assess risk factors and guide risk factor management and lifestyle modification in a remote setting.^[^ ^11^ ^]^ Established risk factors are often assessed only once in a structured way at the time point when AF patients present for the first time in the AF‐clinic (spot‐assessment of risk factors). However, several AF risk factors may show a high visit‐to‐visit or even day‐to‐day variability and lifestyle components such as physical activity, diet and sleep behaviors may be variable over time.^[^ ^11^, ^12^, ^13^, ^14^, ^15^, ^16^, ^17^ ^]^ This visit‐to‐visit or day‐to‐day variability does not just complicate the detection of AF risk factors but may also have a prognostic implication. High visit‐to‐visit variability in risk factors is associated with increased risk of incident new‐onset AF, worse cardiovascular outcome and increased mortality.^[^ ^18^, ^19^, ^20^ ^]^ Hence, assessment of risk factors requires a longitudinal and remote structured monitoring infrastructure (Figure 3). Additionally, longitudinal documentation of risk factors during a risk factor modification program may allow monitoring of the response to the intervention and adaptation and guidance as required to optimize the results.

For the implementation of remote and longitudinal assessment of risk factors and lifestyle components, mHealth applications and technologies such as activity trackers, Bluetooth‐linked balances, blood pressure devices and diary apps to assess diet may provide the required infrastructures. Smartphone apps as well as smartwatches already provide longitudinal information about most lifestyle components and some risk factors. The smartphone app “Health Buddies application”^[^ ^21^ ^]^ or a computer‐animated application designed by Magnani et al.^[^ ^22^ ^]^ resulted in increased adherence to oral anticoagulation and improved quality of life in AF patients. A meta‐analysis of 51 randomized controlled trials demonstarted that compared with usual care, mHealth interventions in diabetes and hypertension management yielded significant mean differences in clinical outcomes including blood pressure, fasting blood glucose and HbA1c control and had positive effects on improving quality of life, satisfaction and self‐efficacy.^[^ ^23^, ^24^ ^]^ However, until now, these apps are almost exclusively patient‐initiated and not implemented in structured patient care pathways. Therefore, most of the apps remain as lifestyle products and have not found their way to clinical implementation.

Another emerging modifiable AF risk factor is sleep apnea.^[^ ^25^ ^]^ Technologies implemented in implantable devices, on‐contact biomotion sensors with actimetry, ballistic sensors or Doppler technology with radar frequencies can remotely monitor breathing during sleep.^[^ ^26^, ^27^ ^]^ Wearable devices with inbuilt pulse oximeters are now also becoming commercially available raising the prospect of routinely measuring the night‐to‐night variability of sleep disordered breathing severity by means of the oxygen desaturation index or determining hypoxic burden more broadly by newer validated algorithms.^[^ ^17^, ^28^ ^]^ Several clinical trials have demonstrated the feasibility of mHealth‐based sleep apnea management compared with a more traditional in‐person care model, suggesting non‐inferiority in terms of adherence to continuous positive airway pressure treatment and compliance as well as functional outcomes such as satisfaction and cost‐effectiveness.^[^ ^29^, ^30^ ^]^

Technologies for longitudinal monitoring of lifestyle components and AF risk factors are available and may support a more complete remote assessment and management of AF patients in the future, once these tools can be implemented in existing clinical pathways or emerging mHealth approaches. In addition to the assessment of risk factors, mHealth infrastructures and apps can also be helpful in applying dedicated in‐app coaching to improve lifestyle and control risk factors by behavioral changes.^[^ ^31^ ^]^ Besides this, telemonitoring can also optimize medication adherence.^[^ ^32^ ^]^

## 4 CLINICAL IMPLEMENTATION OF TELECONSULTATION AND MHEALTH SOLUTIONS IN AN INTEGRATED CARE APPROACH

Integrated care has been recognized as a suitable approach to manage patients with chronic conditions and complex treatments, such as AF, in international guidelines of the ESC for the management of AF.^[^ ^33^, ^34^ ^]^ Implementation of teleconsultation and the use of mHealth solutions should be embedded within this integrated care approach, whilst adhering to the following four fundamental components:

1) The *use of technology* by means of eHealth or mHealth. This aims to support and guide the patient through the care process (eg, patient education and instruction) as well as the treatment team (smart technology to support decision making).^[^ ^35^ ^]^ In fact, such technology solutions should support integrated care in terms of actively involving patients in their care process, collaboration within multidisciplinary teams, and provide guidance in the complex (shared) treatment decisions and coordination of care.

2) Active *involvement of the patient* is promoted through the mHealth solutions. The role of the patient in an mHealth infrastructure is crucial, as the treatment team relies on the patient to use the infrastructure and collect and provide data on vital parameters such as heart rate and rhythm as well as symptoms and potential risk factors. However, before patients can take on such a task, they should be educated and clearly instructed, so they understand what is expected from them.

3) The treatment may be provided by a *multidisciplinary treatment team*. This team consists of cardiologists, nurses, primary care physician and other specialists that might be involved in the management of AF, depending on the individual case. Infrastructure for such an approach might be available in terms of a specialized AF‐clinic,^[^ ^36^ ^]^ where cardiologists and specialized nurses would work closely with the patient, in a face to face setting or via teleconferencing, aiming to improve efficiency and outcomes.^[^ ^37^ ^]^ Communication is key in such teams to assure that all team members understand their role and contribution, and that there is a designated care coordinator. Depending on the context this may be a nurse within the AF‐clinic or administrative staff.

4) The final component of integrated care is the *delivery of comprehensive treatment*. Besides the management of AF (ie, heart rate and/or rhythm control strategy to improve symptoms) alone, it is crucial to determine the potential stroke risk and prescribe appropriate oral anticoagulation accordingly to prevent thromboembolic complications; management of precipitating factors (such as underlying cardiovascular conditions and modifiable risk factors) to reduce the cardiovascular burden to consequently reduce the AF burden.^[^ ^33^, ^38^, ^39^, ^40^ ^]^

**FIGURE 4:**
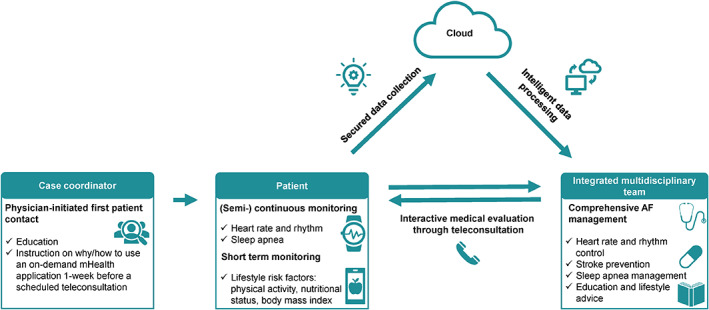
Remote heart rate, rhythm and risk factor assessment by the use of mobile health solutions through teleconsultation

These four fundamentals form the basis of an integrated AF care approach and the use of mHealth solutions through teleconsultations, seamlessly fits within an AF‐clinic as well as with the aims of integrated care: improving outcomes while preventing fragmentation of care. One possible pathway to implement an on‐demand mHealth infrastructure for remote heart rate, rhythm and risk factor assessment to allow comprehensive AF management through teleconsultation is shown in Figure 4.

## 5 AN EXAMPLE OF AN MHEALTH PROJECT TO MANAGE AF PATIENTS THROUGH TELECONSULTATION DURING COVID‐19

**FIGURE 5:**
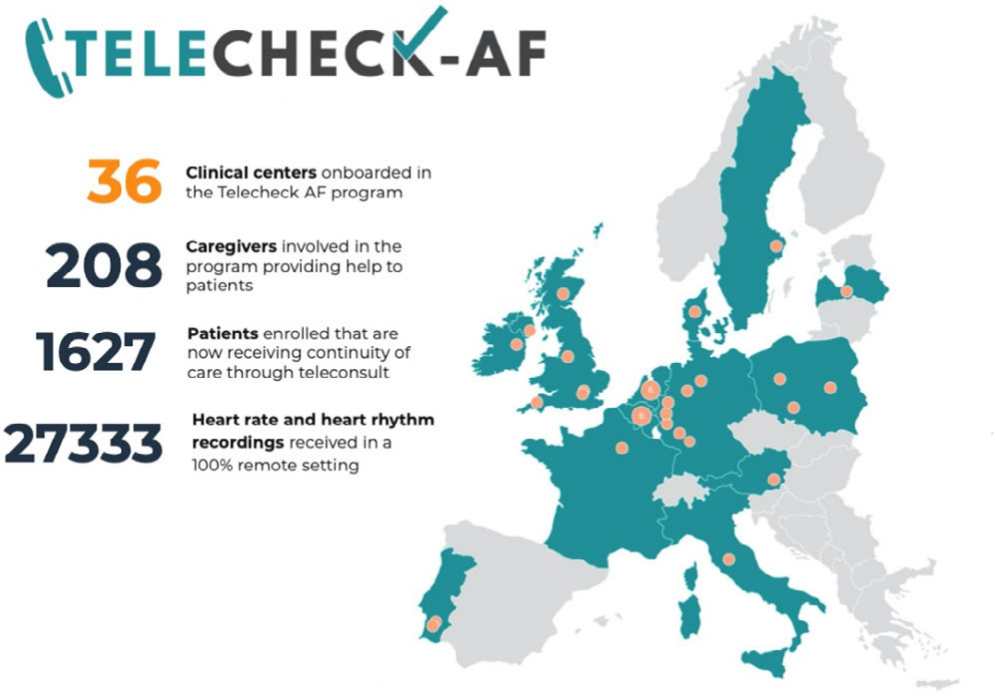
The TeleCheck‐AF project

TeleCheck‐AF is an on‐demand mHealth intervention incorporating an app‐based heart rate and rhythm monitoring infrastructure to allow remote AF management through teleconsultation. During the COVID‐19 pandemic, it was made available in several European centres to keep AF patients out of the hospital (see Figure 5 **)**.^[^ ^39^, ^41^, ^42^ ^]^ TeleCheck‐AF involves a structured teleconsultation (“Tele”) preceded by an app‐based on‐demand heart rate and rhythm monitoring infrastructure (“Check”) to guarantee comprehensive AF management (“AF”).^[^ ^39^ ^]^ The Conformité Européenne (CE)‐marked PPG‐based mobile phone app (www.fibricheck.com) allows semi‐continuous heart rate and rhythm monitoring of AF patients for 7 days prior to and during the teleconsultation. One important advantage of TeleCheck‐AF compared to other systems of telemonitoring is the on‐demand mHealth approach.^[^ ^43^ ^]^ It enables the physicians to use heart rate and rhythm data for treatment decisions and prevents unnecessary data collection which would be the case with continuous long‐term heart rate and rhythm telemonitoring systems (eg, wearables devices or CIEDs), and which need to be managed afterwards requiring work‐intensive and expensive data management infrastructures. Additionally, the on‐demand heart rate and rhythm monitoring approach empowers patients to monitor their vital parameters and self‐manage their condition. Patients are involved in making decisions about measurement time and number of measurements during the day, that depends in particular on the presence of symptoms. Furthermore, the TeleCheck‐AF approach provides crucial information about symptom‐rhythm correlation by simultaneous rhythm and symptom assessment to steer appropriate AF management. The TeleCheck‐AF infrastructure can be combined with other available app‐based risk factor assessment tools, to allow the comprehensive remote assessment and management of AF patients.

## 6 FURTHER CHALLENGES FOR IMPLEMENTATION OF MHEALTH IN CLINICAL PRACTICE

Implementation of mHealth infrastructures require adaptation of existing care coordination and clinical pathways.^[^ ^43^ ^]^ An important element for embedding mHealth in clinical practice is the accessibility of the recordings by other healthcare professionals. For this, a connection with the patients' electronic healthcare record is crucial. This connection facilitates automatic transmission of the recordings from the secured cloud to the electronic healthcare record of the patient and increases the accessibility of the data for other healthcare professionals. Additionally, many AF apps lack scientific validation and are written at excessively high reading‐grade levels challenging users with limited health literacy. Although mHealth solutions for heart rate and rhythm monitoring are clinically established and used, apps for longitudinal risk factor assessment are not available and were therefore not yet incorporated in the TeleCheck‐AF approach. Finally, a multi‐disciplinary effort by regulatory agencies, healthcare organizations, and app stores is required to improve relevance, scientific validity, and readability of AF apps for AF patients.^[^ ^44^ ^]^ Additionally, discussions with insurance companies about reimbursement of mHealth infrastructures and with different stakeholders to agree on security and privacy regulations are initiated in different countries.^[^ ^31^, ^45^ ^]^

## 7 CONCLUSION

Health tools in the management of AF are becoming indispensable in current healthcare. Novel tools are able to remotely assess heart rate and rhythm and incorporate AF risk factor assessment to allow comprehensive AF management through teleconsultation. TeleCheck‐AF is one, but not the only possible solution to improve remote AF care during the COVID‐19 pandemic and will provide insight into the additional value and potential limitations of mHealth strategies to remotely manage AF patients. Such mHealth infrastructures may be well suited within an integrated AF‐clinic, which may require redesign of practice and reform of health care systems.

## CONFLICT OF INTEREST

The authors declare that there is no conflicts of interests.

## Data Availability

Data sharing not applicable to this article as no datasets were generated or analysed during the current study.
